# Differential diagnosis of COVID-19 and influenza

**DOI:** 10.1371/journal.pgph.0000221

**Published:** 2022-07-21

**Authors:** Farrokh Alemi, Jee Vang, Janusz Wojtusiak, Elina Guralnik, Rachele Peterson, Amira Roess, Praduman Jain

**Affiliations:** 1 Department of Health Administration and Policy, College of Health and Human Services, George Mason University, Fairfax, VA, United States of America; 2 Vibrent Health, Inc., Fairfax, VA, United States of America; 3 Department of Global and Community Health, College of Health and Human Services, George Mason University, Fairfax, VA, United States of America; The University of Sydney, AUSTRALIA

## Abstract

This study uses two existing data sources to examine how patients’ symptoms can be used to differentiate COVID-19 from other respiratory diseases. One dataset consisted of 839,288 laboratory-confirmed, symptomatic, COVID-19 positive cases reported to the Centers for Disease Control and Prevention (CDC) from March 1, 2019, to September 30, 2020. The second dataset provided the controls and included 1,814 laboratory-confirmed influenza positive, symptomatic cases, and 812 cases with symptomatic influenza-like-illnesses. The controls were reported to the Influenza Research Database of the National Institute of Allergy and Infectious Diseases (NIAID) between January 1, 2000, and December 30, 2018. Data were analyzed using case-control study design. The comparisons were done using 45 scenarios, with each scenario making different assumptions regarding prevalence of COVID-19 (2%, 4%, and 6%), influenza (0.01%, 3%, 6%, 9%, 12%) and influenza-like-illnesses (1%, 3.5% and 7%). For each scenario, a logistic regression model was used to predict COVID-19 from 2 demographic variables (age, gender) and 10 symptoms (cough, fever, chills, diarrhea, nausea and vomiting, shortness of breath, runny nose, sore throat, myalgia, and headache). The 5-fold cross-validated Area under the Receiver Operating Curves (AROC) was used to report the accuracy of these regression models. The value of various symptoms in differentiating COVID-19 from influenza depended on a variety of factors, including (1) prevalence of pathogens that cause COVID-19, influenza, and influenza-like-illness; (2) age of the patient, and (3) presence of other symptoms. The model that relied on 5-way combination of symptoms and demographic variables, age and gender, had a cross-validated AROC of 90%, suggesting that it could accurately differentiate influenza from COVID-19. This model, however, is too complex to be used in clinical practice without relying on computer-based decision aid. Study results encourage development of web-based, stand-alone, artificial Intelligence model that can interview patients and help clinicians make quarantine and triage decisions.

## Introduction

It is increasingly clear that COVID-19 is becoming an endemic disease and clinicians would need to accurately differentiate it from seasonal influenza and influenza-like-illnesses. A number of existing published studies have contrasted differential diagnosis of COVID-19 and influenza in patients who present at the hospital and for whom laboratory data are available [1, 2]. The current study focuses on the symptoms reported in emergency departments, or clinic visits, prior to hospitalization, when no laboratory data are available. Accurate symptom screening can help clinicians triage community patients to appropriate settings, to order at-home or point-of-care rapid tests, or to suggest the length of quarantine. Our previous study had examined differential diagnosis of COVID-19 using data from China from the early days of the pandemic, with a limited set of symptoms, and under only two scenarios: widespread flu or no flu [3]. The current study expands the possible scenarios, includes more symptoms, and relies entirely on the experiences of patients within the United States.

When a new infection emerges, it is important to quickly clarify its signature presentation and symptoms that can help differentiate it from other diseases. The U.S. Centers for Disease Control and Prevention (CDC) has repeatedly changed the guidance on which symptoms can be used to diagnose COVID-19. At the time of publication of this paper, the CDC listed common symptoms of COVID-19; and provides no guidance on how to weigh these symptoms, either individually or in clusters of symptoms. It provides no guidance on how to differentiate COVID-19 from influenza or influenza-like illness. This study aims to clarify how COVID-19 may be differentiated from influenza based on the symptoms of patients presenting in the community, based on the data collected at home or in other settings (e.g. clinics), but referring to symptoms present prior to any hospitalization.

There are considerable variations in prevalence of respiratory illnesses. During the year 2020, while social distancing was implemented, there were few influenza or influenza-like-illness cases, necessitating for us to rely on data from the years prior to the emergence of COVID-19 [4, 5]. This trend can continue, or influenza can return when schools open. COVID-19 may decline through vaccination, or other scenarios can affect the prevalence of COVID-19 and other respiratory diseases in various locations. Therefore, clinicians would need to be prepared to differentiate respiratory diseases under a variety of scenarios. This study examines differential diagnosis of COVID-19 under 45 different scenarios regarding the spread of COVID-19, influenza, or influenza-like-illnesses.

## Methods

When a pandemic emerges, reliance on existing data sources can accelerate identification of signature symptoms of the new infection. This study relied on two different existing data sources. The first dataset obtained from the U.S. Centers for Disease Control and Prevention (CDC) [6], was collected between March 1, 2019, through September 30, 2020. The data included 3.5 million, laboratory-verified, positive COVID-19 cases. These data provided the cases in this study while the controls for these cases came from a different data source. The second data set was obtained from the Influenza Research Database of the National Institute of Allergy and Infectious Diseases (NIAID) and included 1,814 influenza and 812 influenza-like illness cases reported to the database between January 1, 2000, and December 30, 2018 [7]. This time period provided the most recent data that were available at the time. Both data sets were completely anonymized prior to the authors receiving access.

The two data sources (S1 and S2 Data) used in this study had different definitions of symptoms. Two authors (JV, FA) reviewed those definitions and created a consistent nomenclature across the data sources.

To be included in the study, both COVID-19 cases and influenza/influenza-like illness cases must have reported at least one of the following symptoms: (1) cough, (2) fever, (3) chills, (4) diarrhea, (5) nausea and vomiting, (6) shortness of breath, (7) runny nose, (8) sore throat, (9) myalgia, and (10) headache. Therefore, the study findings are only generalizable to symptomatic COVID-19 patients. In the CDC data, the majority of the COVID-19 patients were either asymptomatic or their symptoms were not reported. Of 3.5 million of the laboratory-confirmed positive COVID-19 cases, 839,288 cases (24%) had reported at least one symptom, hence were included in our analysis. In the influenza and influenza-like illness databases, all listed cases had at least one symptom reported. For patients with at least one symptom, if additional symptom were missing at random, the missing values were assumed to be absent (the mode for the responses).

Symptoms reported in one but not the other database could not be used in the regression equations. It has been noted that COVID-19 presents with non-respiratory symptoms as well (e.g. loss of smell or taste). In those situations, influenza or influenza-like illnesses were not suspected. Only patients presenting with common respiratory infection symptoms across the two databases were included in the analysis.

We constructed models for differentiating COVID-19 from influenza/influenza-like illness under 45 scenarios. These scenarios were constructed from different assumptions about the prevalence of COVID-19, influenza, and influenza-like illness, co-occurring during the same season. In the future, we assumed that the prevalence of COVID-19 will be 2%, 4%, or 6% of the population. The prevalence of influenza, in the future, was assumed to be 0.01%, 3%, 6%, 9%, or 12% of the population. In the future, the prevalence of influenza-like illness was assumed to be 1%, 3.5% or 7% of the population. The combination of these assumptions produced 45 different scenarios, reported in S1 Table. In December 2020, United States had scenario #1 (i.e., 2% COVID-19, 0.01% influenza, and 3.5% influenza-like illness). For each of these 45 scenarios, we randomly sampled 10,000 cases.

In each scenario, to differentiate COVID-19 from influenza or influenza-like illness, we used ordinary logistic regressions. In these regressions, the dependent variable was laboratory-confirmed COVID-19 test results. The independent variables were age, gender, and the 10 symptoms shared across the two databases. Regressions were done with both linear and interaction terms. Interaction terms were organized among age above 30, gender, and binary symptoms, taken in pairs, 3-way, 4-way, and 5-way. In total, there were 5,519 combinations of interaction terms possible.

To reduce the possibility of modeling noise, the models were constructed and tested using 5-fold cross-validation [8]. In particular, for each scenario, 10,000 cases were generated by uniform random sampling from the 2 different datasets, one containing laboratory-confirmed COVID-19 cases with at least one reported symptom, and the other containing laboratory-confirmed influenza/influenza-like illness controls which reported at least one symptom. The proportion of data sampled from each database corresponded to the relative prevalence of the diseases within the 45 scenarios. 80% of these data were randomly set aside for model development and 20% of the data were used for model validation. Random sampling was repeated 5 times and the average accuracy was reported across the 5 samples. The 5-fold cross-validated Area under the Receiver Operating Curves (AROC) was used to report the accuracy of the models under different scenarios.

## Results

S2 Table describes the distribution of cases by age and gender within the three groups from the two data sources. The cases from the two databases were weighted to reflect the age and gender in the United States population.

S3 Table shows how the inclusion of interaction terms affected the accuracy of models created across 45 scenarios. In 5-fold cross-validation, the simple main-effect logistic regression, with no-interaction terms, had AROC of 0.69. As the number of interactions terms increased, the accuracy of the regression models improved. When up to 5-way interaction was included, the regression model had a 5-fold cross-validated AROC of 0.90.

**Fig 1:**
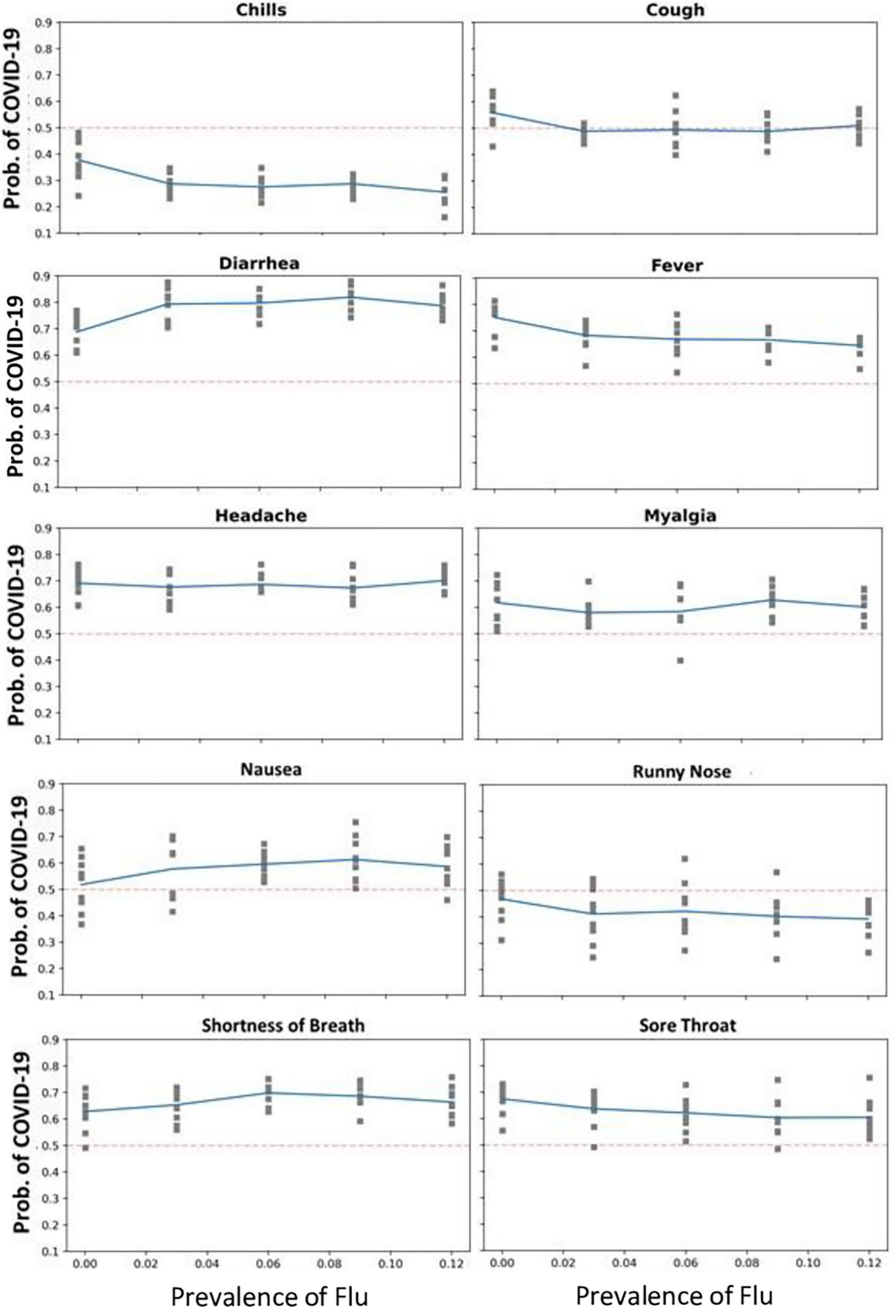
Impact of symptoms on diagnosis of COVID-19 in different scenarios. The red dotted line indicates the point which the symptom switches from predictive of to ruling out COVID-19. The solid blue line indicates the average across scenarios. Square grey markers show impact of symptom in different scenarios.

The diagnostic value of different symptoms (regression coefficients associated with the symptoms) changed in the 45 scenarios. The full list of coefficients of the regression models is presented in S1 File. In 6 out of 10 symptoms, the impact of the symptom was reversed under at least one of the scenarios. A reversal means that a coefficient that was predictive of COVID-19, a positive coefficient, became negative and useful to rule out COVID-19; and vice versa, symptoms predictive of influenza/influenza-like illness switched and became predictive of COVID-19. Fig 1 shows how the impact of symptoms changed with the prevalence of influenza. The X-axis is the prevalence of influenza. The Y-axis is the probability of COVID-19, with at least one symptom present. In Fig 1, grey squares represent impact of symptoms in the 45 scenarios (nine dots per each of five prevalence levels for influenza). When those markers are above the dotted line, then a symptom is predictive of COVID-19; and otherwise, it is not. For example, the plot for cough shows how cough changed as a predictor of COVID-19 in different scenarios. When influenza was prevalent (0.06%—a common situation during the historical peak of influenza season), cough ruled out COVID-19. When influenza was absent, cough indicated the presence of influenza. The value of cough in diagnosing COVID-19 depended on the prevalence of influenza.

Furthermore, regression coefficients for different interaction terms suggested that the differentiation of COVID-19 and influenza/influenza-like illness was impacted by age. In 52.65% of scenarios, the impact of a symptom combination reversed when the age group was switched from 20–29 to 50–59 years old. For example, consider the scenario in which the prevalence of COVID-19 is 4%, influenza is 9%, and influenza-like illness is 3.5%. In this scenario, it is informative to look at the diagnostic value of a combination of fever and sore throat on odds of having COVID-19. In the age group of 20–29 years old, that combination of symptoms reduced the odds of having COVID-19 (Odds of 0.18). In individuals in the age group of 50 to 59 years old, the same set of symptoms increased the odds of having COVID-19 (Odds of 2.02). These findings are consistent with others in the literature, suggesting that COVID-19 presentation differs for various age groups [9].

## Discussion

There are a number of limitations in this study which should be considered before evaluating the findings. This study has focused on COVID-19 cases that present with respiratory symptoms. Not all SARS-CoV-2 infections have presented with symptoms. Furthermore, not all symptomatic COVID-19 patients present with respiratory symptoms. The models constructed and validated in this study would not be applicable to asymptomatic individuals or those without respiratory symptoms.

Another limitation of this study is related to the fact that this study relied on two sources of data, collected at different time periods. COVID-19 cases used in our analysis occurred in 2019 and 2020. Adherence to masking and social distancing, especially during the early days of COVID-19 pandemic had reduced the number of influenza cases in 2020 to nearly zero [5]. It was not possible to find sufficient number of influenza cases in 2019 or 2020. Thus, this study relied on influenza and influenza-like illness data from the years prior to 2019. The collection of data over two different time periods may introduce errors in our predictions, especially if the symptoms had changed over those time periods.

This study was done before the emergence of Omicron and Delta variants of SARS-CoV-2. At the time of the publication of this paper, there were reports that different variants of the novel coronavirus may present with different symptoms [10]. Additional data are needed to establish with more certainty how different variants of SARS-CoV-2 affect differential diagnosis of COVID-19 from influenza. Finally, this study was limited because data on disease control measures, individual risk-taking behaviors, or exposures were not available from the data sources used in this study. Assessment of known or suspected exposures of patients is an important measure used by clinicians to aid in differential diagnosis. Clinicians often diagnose a sibling’s respiratory illness because of the exposure at home.

Despite these limitations, the study suggests the complexity of differentiating COVID-19 from influenza/influenza-like illness. Published literature [11], the CDC’s guidance [12], and current clinical practices rely on the use of common features of COVID-19 to screen and triage patients. During the pandemic, many clinics have been screening patients with a list of common symptoms of COVID-19 by asking, at the point of care, if they had recently experienced any of those symptoms. Anyone having a single symptom on the list would be triaged to COVID-19 designated areas [13]. These efforts treat influenza cases as COVID-19 cases, sending both sets of patients to the same waiting areas, hence increasing the risk of overlapping infections (i.e., influenza patients getting infected with COVID-19). The study found that symptom screening should consider different symptoms at various ages. The coefficients of logistic regressions for symptoms changed in different ages. The same symptom which indicated COVID-19 in older patients, indicated influenza in younger patients.

Symptom screening should focus on clusters and not individual symptoms. Models based on pairs of symptoms were less accurate (AROC = 0.69) than models based on 5-way interaction between symptoms (AROC = 0.90), suggesting the importance of clusters of symptoms. Clinicians cannot rely on simple rules for diagnosing COVID-19 and would need to examine combinations of symptoms.

To differentiate COVID-19 from influenza and influenza-like illness, symptom screening should consider the prevalence of the pathogens. The impact of symptoms on diagnosis of COVID-19 changed under different scenarios. In majority of scenarios, the impact of at least one symptom cluster reversed. If the symptom was originally indicative of COVID-19, under different assumptions of prevalence of pathogens, it reversed direction and now ruled-out COVID-19.

The 5-fold cross-validated AROC associated with differentiating COVID-19 from influenza based on their associated symptoms was high (90%). This level of accuracy is high enough to be clinically relevant. At the same time, the models constructed in this study are complex and present challenges in their applicability to clinical practice. Currently, the CDC’s website states that it is not possible to differentiate COVID-19 from other respiratory diseases based on the symptoms alone “because some of the symptoms of flu, COVID-19, and other respiratory illnesses are similar.” [14]. While no simple rule exists for differentiating influenza from COVID-19, this study has shown that it is possible to do so, using 45 models of a large number of symptom combinations. These complex models are not applicable in clinical practice unless computer aids are organized to interview patients and report their likely diagnoses to clinicians.

The sheer number of symptom combinations, assumptions of prevalence of pathogens, and age-symptom combinations greatly exceeds the number of items that any clinician can keep in mind. The complexity of the inference tasks suggests the need for a decision aid that can assist clinicians in making COVID-19 diagnoses more accurately and to allow for better symptom screening in the community. Such tool could automatically account for the prevalence of COVID-19, influenza, and influenza-like illness based on the geographic location of the user; select logistic regression model that is appropriate for the location of the individual; and predict individual’s odds of having COVID-19. Ideally, such web tool should report the probability of COVID-19 using real-time spatial-temporal prevalence of respiratory infections, and report directly to patients, and, through patients, to their clinicians. Other investigators have developed real-time access to forecasting respiratory infections [15].

## Conclusion

The models developed in this study establish how one can differentiate COVID-19 from influenza, albeit only if a computerized decision aid could interview the patient and calculate the probability of a likely infection. Such method of assessment and triage would be helpful if access to at-home COVID-19 tests were limited, as experienced in the United States for some time [16], and as it continues to be limited in many low- and moderate-income countries [17–19].

## Supporting information

S1 File Regression coefficients. (ZIP)

S1 Table Hypothetical scenarios for prevalence of COVID-19, influenza, and influenza-like illness. (DOCX)

S2 Table Demographics of COVID-19 cases and controls. (DOCX)

S3 Table 5-fold cross-validated accuracy of regression models. (DOCX)

S1 Data Symptom-mapping. (TXT)

S2 Data Symptom-mapping. (TXT)
