# Supplementary material for: Differential diagnosis of COVID-19 and influenza
Source: PLOS Glob Public Health. 2022 Jul 21;2(7):e0000221. doi: 10.1371/journal.pgph.0000221 (PMC10021438; doi:10.1371/journal.pgph.0000221)
Supplement: S1 Table — (DOCX) [file pgph.0000221.s002.docx]

|  | **COVID-19** | **Flu-Like** | **Flu** |  | **#** | **COVID-19** | **Flu-Like** | **Flu** |  | **#** | **COVID-19** | **Flu-Like** | **Flu** |
| --- | --- | --- | --- | --- | --- | --- | --- | --- | --- | --- | --- | --- | --- |
| **1** | 2% | 1% | 0.01% |  | **16** | 2% | 1% | 0.01% |  | **31** | 2% | 1% | 0.01% |
| **2** | 2% | 1% | 3% |  | **17** | 2% | 1% | 3% |  | **32** | 2% | 1% | 3% |
| **3** | 2% | 1% | 6% |  | **18** | 2% | 1% | 6% |  | **33** | 2% | 1% | 6% |
| **4** | 2% | 1% | 9% |  | **19** | 2% | 1% | 9% |  | **34** | 2% | 1% | 9% |
| **5** | 2% | 1% | 12% |  | **20** | 2% | 1% | 12% |  | **35** | 2% | 1% | 12% |
| **6** | 2% | 4% | 0.01% |  | **21** | 2% | 4% | 0.01% |  | **36** | 2% | 4% | 0.01% |
| **7** | 2% | 4% | 3% |  | **22** | 2% | 4% | 3% |  | **37** | 2% | 4% | 3% |
| **8** | 2% | 4% | 6% |  | **23** | 2% | 4% | 6% |  | **38** | 2% | 4% | 6% |
| **9** | 2% | 4% | 9% |  | **24** | 2% | 4% | 9% |  | **39** | 2% | 4% | 9% |
| **10** | 2% | 4% | 12% |  | **25** | 2% | 4% | 12% |  | **40** | 2% | 4% | 12% |
| **11** | 2% | 7% | 0.01% |  | **26** | 2% | 7% | 0.01% |  | **41** | 2% | 7% | 0.01% |
| **12** | 2% | 7% | 3% |  | **27** | 2% | 7% | 3% |  | **42** | 2% | 7% | 3% |
| **13** | 2% | 7% | 6% |  | **28** | 2% | 7% | 6% |  | **43** | 2% | 7% | 6% |
| **14** | 2% | 7% | 9% |  | **29** | 2% | 7% | 9% |  | **44** | 2% | 7% | 9% |
| **15** | 2% | 7% | 12% |  | **30** | 2% | 7% | 12% |  | **45** | 2% | 7% | 12% |

**S1 Table**. **Hypothetical Scenarios for Prevalence of COVID-19, Influenza, and Influenza-Like Illness**
