# Supplementary material for: Differential diagnosis of COVID-19 and influenza
Source: PLOS Glob Public Health. 2022 Jul 21;2(7):e0000221. doi: 10.1371/journal.pgph.0000221 (PMC10021438; doi:10.1371/journal.pgph.0000221)
Supplement: S2 Table — (DOCX) [file pgph.0000221.s003.docx]

**S2 Table. Demographics of COVID-19 Cases and Controls**

|  | **COVID-19 Cases** | **Influenza**  **as Controls** | **Influenza-like Illness  as Controls** |
| --- | --- | --- | --- |
| Source of Data | CDC COVID-19 Case Surveillance Data | Influenza Research Database (NIAID) | Influenza Research Database (NIAID) |
| Number of Cases | 839,288 | 1,814 | 812 |
| Percent Positive COVID-19 | 100% | 0% | 0% |
| Percent with at least 1 Respiratory Symptoms | 100% | 100% | 100% |
| Average age in Years^+^ | 41.14 | 33.36 | 27.35 |
| Percent Male^+^ | 47.00% | 46.40% | 38.90% |
| Distribution of Symptoms (%) |  |  |  |
| Chills | 29.39 | 20.26 | 20.27 |
| Cough | 40.98 | 36.60 | 36.62 |
| Diarrhea | 6.76 | 14.94 | 14.91 |
| Fever | 35.31 | 31.37 | 31.39 |
| Headache | 23.00 | 29.34 | 29.32 |
| Myalgia | 15.31 | 28.44 | 28.40 |
| Nausea/Vomiting | 13.13 | 9.02 | 9.03 |
| Runny Nose | 27.93 | 6.44 | 6.49 |
| Shortness of Breath | 12.41 | 18.54 | 18.52 |
| Sore Throat | 23.71 | 17.33 | 17.35 |
