# Supplementary material for: Differential diagnosis of COVID-19 and influenza
Source: PLOS Glob Public Health. 2022 Jul 21;2(7):e0000221. doi: 10.1371/journal.pgph.0000221 (PMC10021438; doi:10.1371/journal.pgph.0000221)
Supplement: S3 Table — (DOCX) [file pgph.0000221.s004.docx]

**S3 Table. 5-Fold Cross-Validated Accuracy of Regression Models.**

| **Type of Logistic Regression Model** | **Number of Scenarios**  **(Sample Size)** | **Average AROC (Standard Deviation)** | **Minimum AROC** | **Maximum AROC** |
| --- | --- | --- | --- | --- |
| Main Effects Only | 45 (45,000) | 0.69 (0.02) | 0.64 | 0.73 |
| Up to 2-Way Interactions | 45 (45,000) | 0.69 (0.02) | 0.63 | 0.74 |
| Up to 3-Way Interactions | 45 (45,000) | 0.85 (0.02) | 0.80 | 0.90 |
| Up to 4-Way Interactions | 45 (45,000) | 0.89 (0.02) | 0.84 | 0.93 |
| Up to 5-Way Interactions | 45 (45,000) | 0.90 (0.02) | 0.84 | 0.94 |

**Notes:** AROC stands for Area under the Receiver Operating Curve. Regressions were done in randomly sampled 45 Scenarios. Response variable was COVID-19 test results. Independent variables were age, gender, and 10 symptoms common in respiratory infections.
